# 15-year incidence of diabetic ketoacidosis at onset of type 1 diabetes in children from a regional setting (Auckland, New Zealand)

**DOI:** 10.1038/srep10358

**Published:** 2015-05-19

**Authors:** Craig Jefferies, Samuel W. Cutfield, José G. B. Derraik, Jignal Bhagvandas, Benjamin B. Albert, Paul L. Hofman, Alistair J. Gunn, Wayne S. Cutfield

**Affiliations:** 1 Starship Children’s Hospital, Auckland District Health Board, Auckland, New Zealand; 2 Liggins Institute, University of Auckland, Auckland, New Zealand; 3 Department of Physiology, University of Auckland, Auckland, New Zealand; 4 Gravida: National Centre for Growth and Development, Auckland, New Zealand

## Abstract

We assessed the incidence of diabetic ketoacidosis (DKA) in children aged <15 years with newly diagnosed type 1 diabetes mellitus (T1DM) in the Auckland Region (New Zealand) in 1999–2013, in a retrospective review of a complete regional cohort. DKA and its severity were classified according to ISPAD 2014 guidelines. Of 730 children presenting with new-onset T1DM over the 15-year time period, 195 cases had DKA of any severity (27%). There was no change in the incidence of DKA or the proportion of children with severe DKA at presentation. The incidence of DKA among children aged <2.0 years (n = 40) was 53% compared to 25% for those aged 2–14 years (n = 690; p = 0.005). In children aged 2–14 years, increasing age at diagnosis was associated with greater likelihood of DKA at presentation (p = 0.025), with the odds of DKA increasing 1.06 times with each year increase in age. Non-Europeans were more likely to present in DKA than New Zealand Europeans (OR 1.52; p = 0.048). Despite a consistent secular trend of increasing incidence of T1DM, there was no reduction in the incidence of DKA in new-onset T1DM in the Auckland Region over time. Thus, it is important to explore ways to reduce DKA risk.

The most serious complication in newly diagnosed cases with type 1 diabetes mellitus (T1DM) is diabetic ketoacidosis (DKA), the leading cause of death in children with T1DM^1^. DKA is preceded by a variable period of symptomatic diabetes characterised by weight loss, polyuria and polydipsia. Early insulin replacement prevents DKA, thus the presence and severity of DKA is largely a consequence of delay in diagnosis and initiation of insulin therapy^2^.

Children with DKA often require prolonged hospitalisation or intensive care. Neuroimaging reveals that a high proportion of patients with DKA at T1DM-onset have evidence of some brain swelling^3^. Although severe swelling is uncommon, it is associated with 20–30% mortality^4^,^5^. Further, DKA is associated with acute reductions of brain volumes, followed by long-term impairment of IQ and short-term memory^3^,^6^,^7^.

Reported risk factors for DKA at presentation include young age, minority ethnicity, poor access to medical care, absence of first-degree relatives with T1DM, and lack of medical insurance^8^. Recent meta-analyses suggest that a higher background incidence of T1DM is associated with a lower risk of DKA in children with newly diagnosed T1DM^2^,^8^,^9^. Although there has been a progressive worldwide increase in the incidence of T1DM^10^,^11^, there is conflicting evidence on the associated effects on DKA rates. While some longitudinal studies found this increase to be associated with reduced incidence of DKA^12^,^13^, others have not^14^,^15^,^16^.

New Zealand has a social security system that provides medical care free of charge. As a result, income is not a direct impediment for take-up of medical care, and the associated costs of T1DM to patients’ families are minimal. We have previously shown that the incidence of T1DM has increased over the last three decades in Auckland, in line with worldwide trends^11^. Auckland is New Zealand’s major urban centre, and its population is ethnically heterogeneous^17^. Previous cross-sectional data from this urban regional service suggest that the frequency of DKA fell from 63% in 1988-89 to 42% in 1995-96^18^. However, there have been significant changes in medical services over this extended interval that may have contributed to this improvement.

In the present study, we aimed to determine: i) whether the incidence of DKA at T1DM-onset in children under 15 years in the Auckland Region has changed over the last 15 years (1999–2013); ii) whether the risk of DKA was associated with specific factors, such as age, sex, socioeconomic status and ethnicity.

## Methods

### Ethics approval

Ethics approval to conduct this study was granted by the Auckland District Health Board Research Review Committee (study number A + 5475, NTX/12/EXP/076). All procedures followed were in accordance with the ethical standards of the responsible committees. Written or verbal informed consent was not required, as this study involved an audit of routine clinical practice.

### Participants

Data were collected on all children aged <15 years diagnosed with T1DM and residing within the Auckland Region between 1 January 1999 and 31 December 2013 from the Starship Children’s Hospital Diabetes Database (Starbase). Additional information was obtained from hospital records as required. The Paediatric Diabetes and Endocrinology Service at Starship Children’s Hospital provides specialist care for a total population of approximately 1.5 million, with case ascertainment levels over 95%^19^.

T1DM was diagnosed based on clinical features. All patients had elevated blood glucose at presentation (a random measurement of >11.1 mmol/l and/or fasting blood glucose >7.1 mmol/l) and presented with classical symptoms. In addition, all patients met at least one of the following criteria: a) DKA; b) presence of pre-T1DM associated antibodies (glutamic acid decarboxylase, islet antigen 2, islet cell, or insulin autoantibodies); or c) on-going requirement for insulin therapy. Subjects with type 2 diabetes, monogenic or other forms of diabetes (e.g. associated with cystic fibrosis) were excluded from this study.

### Study parameters

Demographic and anthropometric data were collected at diagnosis. Height was measured to the nearest 0.1 cm using a Harpenden stadiometer (Holtain, Crosswell, UK). Weight was measured to the nearest 0.1 kg, with the participant in light clothing, by electronic scales. Body mass index standard deviation scores (BMI SDS) were calculated based on British 1990 growth reference data^20^.

Ethnicity was recorded by self-report using a prioritised system, such that if multiple ethnicities were selected, the patient was assigned to a single ethnicity, following a hierarchical classification of Maori, Pacific Islander, Other, and then New Zealand European^17^. “Other” included Indian, South-East Asian, African and Middle Eastern ethnicities. Socioeconomic status was determined using the New Zealand Index of Deprivation 2006 (NZDep2006), a geo-coded deprivation score derived from current residential address^21^.

Bicarbonate and pH were measured from predominantly venous samples. HbA1c was measured by high-performance liquid chromatography analysis (Bio-Rad Laboratories, CA, USA). DKA occurrence was assessed by blood gases using pH and bicarbonate. DKA severity was categorized according to ISPAD 2014 guidelines^22^, as mild (venous pH < 7.3 or bicarbonate <15 mmol/l), moderate (pH < 7.2 or bicarbonate <10 mmol/l), or severe (pH < 7.1 or bicarbonate <5 mmol/l). Autoantibodies to glutamic acid decarboxylase and tyrosine phosphatase-like protein were measured using ELISA kits (RSR Ltd, Cardiff, UK).

### Statistical analysis

Baseline data were assessed using one-way ANOVA or non-parametric Kruskal-Wallis, while sex ratio and ethnic composition data were compared with chi-square tests. The yearly incidence of DKA was assessed as the percentage of all patients newly diagnosed with T1DM who met the criteria for DKA at presentation. Possible changes in incidence and in biochemical blood parameters over the 15-year period were analysed using Poisson regression.

Among subjects who had DKA at diagnosis, ordinary logistic regressions were carried out to assess whether any independent factors (socioeconomic status, sex, ethnicity, and age at diagnosis) were associated with increasing DKA severity. Binary logistic regressions were used to assess whether any of the independent factors were associated with the likelihood of DKA at the time of diagnosis of T1DM throughout the study period.

Linear regression models were used to examine possible associations between the independent factors listed above with glucose, bicarbonate, pH, and HbA1c measurements at diagnosis. In order to assess whether changes differed among age groups, data were also analysed separately for children <2 years and those aged 2-14 years. Possible seasonal variations in DKA incidence were assessed using binary logistic regressions (as described above) and chi-square tests; note that Southern Hemisphere seasons were defined on meteorological criteria^23^. Statistical analyses were carried out using SAS version 9.3 (SAS Institute Inc. Cary NC, USA) and Minitab version 16 (Pennsylvania State University, State College, PA, USA). All statistical tests were two-tailed and maintained at a 5% significance level.

## Results

A total of 955 subjects were diagnosed with diabetes over the study period. 104 were either aged ≥15 years or did not reside in the Auckland Region, 90 were cases of type 2 diabetes, and 31 were classified as “other” (e.g. monogenic of cystic fibrosis-related diabetes). Thus, over the 15-year period, 730 children aged <15 years were diagnosed with new-onset T1DM. Of these, 195 (27% of cases) had DKA at diagnosis, of whom 92 were classified as mild (47%), 52 as moderate (27%), and 51 as severe (26%). Note that the age at diagnosis among those in DKA increased by approximately 0.22 years per year, from 7.2 years in 1999–2000 to 9.6 years in 2012–2013 (p = 0.002). However, this was a likely reflection of an overall increase in the average age at T1DM diagnosis, which also rose over the study period (p = 0.037, as previously reported^11^).

**Figure 1:**
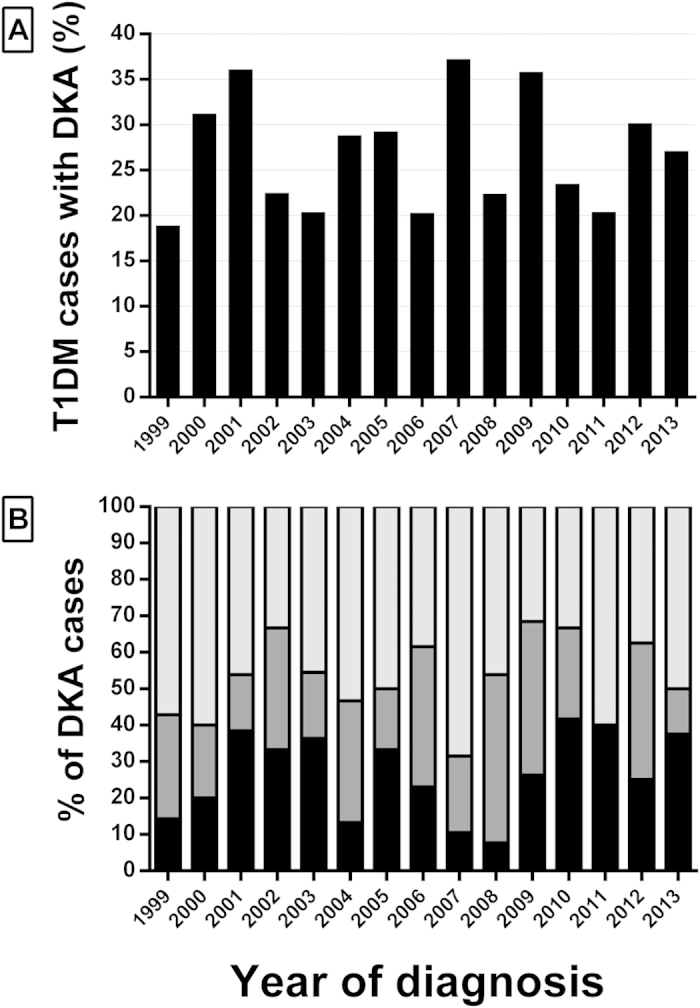
**A**) Percentage of subjects with DKA at T1DM diagnosis in the Auckland Region (New Zealand) from 1999 to 2013; **B**) Proportion of subjects with mild (light grey), moderate (dark grey), and severe (black) DKA at T1DM diagnosis.

The yearly proportion of patients in DKA varied from 19% to 37% (Fig. 1A), but there was no overall change in the incidence of DKA (p = 0.81). When the cohort was assessed as a whole, DKA was not associated with age at diagnosis (p = 0.94), sex (p = 0.09), ethnicity (p = 0.10), or socioeconomic status (p = 0.59). There was also no seasonal variation in DKA incidence (data not shown).

Overall, older age at diagnosis was associated with higher HbA1c (β = 0.296; p < 0.0001) but lower glucose concentrations (β = −0.258; p = 0.018). Non-Europeans had higher HbA1c values (12.7 vs. 11.6%; p = 0.001) and higher glucose concentrations (28.8 vs. 25.9 mmol/l; p = 0.003) than New Zealand Europeans.

Children with DKA displayed similar age at diagnosis, sex ratio, ethnic composition, and socioeconomic status to those who were not in DKA (Table 1), but lower BMI by a mean of 0.33 SDS (Table 1). As expected, children in DKA had lower pH and bicarbonate, and higher glucose and HbA1c levels than patients who were not in DKA (Table 1).

### DKA severity

The proportion of children in severe DKA did not change over the study period (p = 0.48), despite marked variation from year to year (Fig. 1B). The severity of DKA at T1DM diagnosis was unaffected by age or socioeconomic status (Table 2). However, non-Europeans were more likely to have milder DKA at diagnosis than New Zealand Europeans (odds ratio (OR) 1.93; p = 0.032), and there was a possible trend for boys to have milder DKA than girls (OR 1.70; p = 0.056) (Table 2).

### Age bands

40 children were aged less than 2 years at diagnosis of T1DM. The incidence of DKA in this age group was 53% (21/40) compared to 25% (174/690) for children aged 2–14 years (p = 0.005). Among those with DKA, 38% of children aged less than 2 years had severe DKA in comparison to 25% of older children (p = 0.34). In this youngest group, HbA1c levels were considerably higher in non-Europeans than in New Zealand Europeans (12.1 vs 8.7%; p = 0.014). In addition, lower socioeconomic status was associated with higher glucose concentrations (p = 0.022).

**Figure 2:**
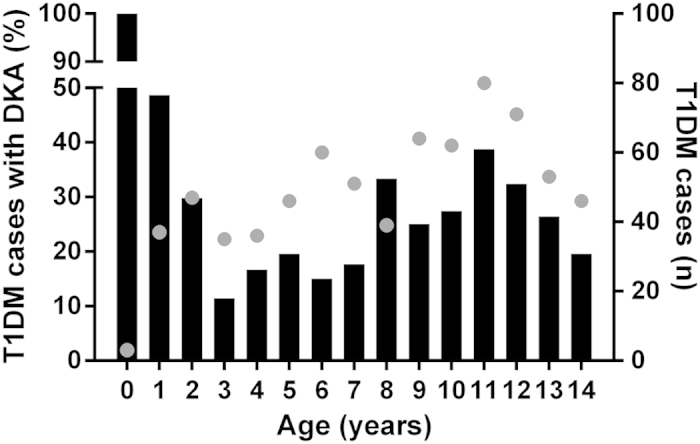
Percentage of subjects with DKA according to age at T1DM diagnosis (black bars on left y axis), and total number of new T1DM cases in the Auckland Region (New Zealand) from 1999 to 2013 (gray dots on right y axis).

Among children aged 2 to 14 years (n = 690), increasing age at diagnosis was associated with greater likelihood of being in DKA at presentation (β = 0.058; p = 0.025; Fig. 2), with the odds of DKA increasing 1.06 times with each year increase in age (95% confidence interval 1.01–1.12). As a result, older age was associated with lower pH (β = −0.005; p = 0.0005) and higher HbA1c levels (β = 0.230; p < 0.0001). In this age group, non-Europeans were more likely to be in DKA at presentation than New Zealand Europeans (OR 1.52; p = 0.048), but were more likely to have mild DKA (OR 2.07; p = 0.026). Non-Europeans also had higher HbA1c levels (12.8 vs 11.7%; p = 0.002) and higher glucose concentrations (28.9 vs 25.7 mmol/l; p = 0.002).

Girls aged 2–14 years had lower bicarbonate concentrations (19.0 vs 20.2 mmol/l, respectively; p = 0.027) and higher HbA1c levels (12.6 vs 11.9%, respectively; p = 0.015) than boys. In addition, girls were less likely to have mild DKA than boys (OR 0.46; p = 0.011).

## Discussion

In this complete regional population of children newly diagnosed with T1DM, an average of 27% were in DKA at presentation over a 15-year period, but with considerable variation in the annual incidence. The age at diagnosis of DKA increased progressively, reflecting the increased average age at presentation with T1DM. Furthermore, consistent with previous studies^24^,^25^,^26^, the risk of DKA was considerably higher in children aged less than 2 years.

The literature suggests that a higher overall rate of T1DM is associated with a lower risk of DKA at presentation with new-onset T1DM^8^. For example, a Finnish study observed a relative reduction in the incidence of DKA over a 20-year period, particularly among children < 5 years of age^12^. In contrast, and consistent with the present study, a large prevalence study of > 14,000 children from 106 centres in Germany and Austria observed no change in DKA incidence or severity over a similar period (1995-2007)^14^. Further, recent nationwide study of 1299 children aged < 15 years with T1DM in France found a continuing high rate of DKA (43.9%)^27^. Similarly, the SEARCH for Diabetes in Youth Study found that the incidence of DKA (in 5615 cases of T1DM from 0 to 19 years of age) was high and stable from 2002 to 2010 in 5 American centers^15^.

Over the past 20 years in the greater Auckland region, there has been an apparent improvement and then stabilisation of DKA incidence from 64% in 1988–9, to 42% in 1995–6, and 27% in the present study, with a concomitant reduction in severity of DKA^18^. This is less than the rate of 38% reported in Western Sydney (Australia) in 1998 to 2010^28^. Further, previous data from 11 European centres^9^ suggest that the current risk of DKA in Auckland is broadly consistent with its incidence of T1DM. The reasons for the lack of further reduction in the risk of DKA over the last 15 years are unclear. The inverse relationship between rate of T1DM and risk of DKA at onset between countries has been attributed to improved awareness of T1DM amongst families and medical professionals as the condition becomes more common. However, the historical improvement in risk of DKA in Auckland may have been partly related to other factors, such as the development of a specialist Paediatric Diabetes Service for the region, as well as technological advances (e.g. the widespread availability of glucose meters). The lack of further change in risk of DKA in the Auckland region since these changes were introduced suggests that factors beyond ‘awareness’ are important contributors to risk of DKA.

The risk factors for DKA at presentation identified in previous studies include younger age, minority race/ethnicity, lower income, lack of private health insurance, and lack of family history of T1DM^2^,^15^. The present study is broadly consistent with this background. The youngest presenting patients (aged < 2 years) constituted a relatively small group (5.5% overall), but had a nearly 2-fold greater rate of DKA compared to children aged 2 to 14 years. The most likely reason for the high rate of DKA in toddlers is that it is simply more difficult to recognize polyuria or polydipsia in very young children, who for example, may be still in nappies. Interestingly, amongst the much larger group of children older than 2 year of age (approximately 95% of the cohort), there was a significant overall increase in risk of DKA with greater age, despite the lower average risk compared to toddlers. In contrast with the known increase in risk of secondary DKA in adolescents, the rate of DKA in the present cohort was maximal around 11 years of age. Speculatively, this may reflect greater day to day involvement in toileting and provision of water for younger children by their parents, and thus earlier detection of symptoms, balanced by greater self-awareness and earlier reporting of symptoms amongst children older than 11 years.

Non-Europeans aged 2 to 14 years were more likely to be in DKA than New Zealand Europeans. The reasons are unclear, since socioeconomic status did not appear to be a factor, most likely as a result of the universal access to health care in New Zealand. We have previously shown that the secular trend to increased T1DM in Auckland was similar in New Zealand Europeans and Non-Europeans^11^. Thus, this cannot explain the increased risk of DKA at diagnosis of T1DM. Potentially, it may relate to awareness of T1DM amongst ethnic minorities or cultural-related factors (such as engagement with medical practitioners).

A limitation of the present study is that we were unable to analyse the presence or not of first-degree relatives with T1DM. Previous studies have found a greatly reduced incidence of DKA in children with a first-degree relative with T1DM^2^,^29^, supporting the benefits of increased awareness in the prevention of DKA at diabetes diagnosis. In addition, it was not possible to identify any pre-admission visits to primary care prior to T1DM diagnosis.

Nonetheless, our findings, viewed in the context of the lower rate of DKA presentation with greater incidence of T1DM demonstrated in meta-analyses, suggest that increasing community awareness is likely to be central to further reduce the incidence of DKA at presentation^8^. Unfortunately, there is contradictory evidence for public education campaigns to improve awareness of the symptoms of T1DM. The ‘Parma campaign’ delivered posters, promoting the link between enuresis, polyuria and diabetes to schools, parents, and paediatric practices, and was associated with a reduction in DKA incidence at diagnosis from 78% to 12.5% over two years^30^. However, subsequent studies have had mixed results^31^,^32^,^33^.

In conclusion, there has been no reduction in the risk of DKA in children with newly diagnosed T1DM over a 15-year period in the greater Auckland region of New Zealand, despite publically supported health care and a secular trend of increased incidence of T1DM^11^. This suggests that direct action to improve community and medical awareness of T1DM is needed to further reduce the rate of DKA at the time of diagnosis.

## Additional Information

**How to cite this article**: Jefferies, C. *et al.* 15-year incidence of diabetic ketoacidosis at onset of type 1 diabetes in children from a regional setting (Auckland, New Zealand). *Sci. Rep.* **5**, 10358; doi: 10.1038/srep10358 (2015).

## Figures and Tables

**Table 1:** Demographics at presentation of children newly diagnosed with type 1 diabetes mellitus, comparing participants with diabetic ketoacidosis (DKA) versus those who did not have DKA. Where appropriate, data are means ± standard deviations.

	**No DKA**	**DKA**	**p-value**
N	535	195	
Age at diagnosis (years)	8.6 ± 3.8	8.6 ± 4.1	0.94
Sex ratio (males)	55%	48%	0.08
Ethnicity			0.40
New Zealand European	68%	63%	
Maori	9%	9%	
Pacific Islander	12%	14%	
Other	11%	14%	
Socioeconomic status (NZDep2006)	4.9 ± 2.8	4.9 ± 2.9	0.83
BMI SDS	0.61 ± 1.31	0.28 ± 1.42	0.004
Biochemical parameters			
pH	7.38 ± 0.04	7.16 ± 0.12	<0.0001
Bicarbonate (mmol/l)	22.7 ± 4.2	11.2 ± 6.4	<0.0001
Glucose (mmol/l)	25.5 ± 9.8	30.6 ± 13.1	<0.0001
HbA1c (%)	11.9 ± 5.4	13.0 ± 2.7	<0.0001

**Table 2:** Demographics at presentation of children with diabetic ketoacidosis (DKA) at diagnosis of type 1 diabetes mellitus, comparing participants according to DKA severity (defined as per ISPAD 2014 guidelines^22^). Different superscript letters indicate statistically significant differences at p < 0.05. Where appropriate, data are means ± standard deviations.

	**Mild DKA**	**Moderate DKA**	**Severe DKA**
N (proportion of cohort)	92 (47%)	52 (27%)	51 (26%)
Age at diagnosis (years)	8.8 ± 3.9	8.8 ± 4.0	8.1 ± 4.6
Sex ratio (males)	52%^A^	56%^A^	31%^B^
Ethnicity (New Zealand European)	53%^A^	71%^B^	71%^B^
Socioeconomic status (NZDep2006)	5.3 ± 3.0	4.5 ± 2.8	4.7 ± 2.6
BMI SDS	0.27 ± 1.26	0.39 ± 1.44	0.17 ± 1.67
Biochemical parameters			
pH	7.26 ± 0.03^A^	7.16 ± 0.03^B^	6.99 ± 0.08^C^
Bicarbonate (mmol/l)	14.8 ± 4.5^A^	9.4 ± 6.3^B^	6.9 ± 6.2^C^
Glucose (mmol/l)	30.5 ± 11.9	29.0 ± 11.5	32.6 ± 16.2
HbA1c (%)	13.1 ± 1.9	12.4 ± 1.9	13.6 ± 4.4
